# Dynamic brain mechanisms supporting salient memories under cortisol

**DOI:** 10.1126/sciadv.adz4143

**Published:** 2025-12-10

**Authors:** Yuye Huang, David O’Connor, Bailey B. Harris, Rajita Sinha, R. Todd Constable, Elizabeth V. Goldfarb

**Affiliations:** ^1^ Department of Psychological & Brain Science, Johns Hopkins University, Baltimore MD 21218, USA.; ^2^ Icahn School of Medicine, Mount Sinai, NY 10029, USA.; ^3^ Department of Psychology, University of California Los Angeles, Los Angeles, CA 90095, USA.; ^4^ Department of Psychiatry, Yale University, New Haven, CT 06511, USA.; ^5^ Department of Diagnostic Radiology, Yale University, New Haven, CT 06510, USA.; ^6^ Department of Psychology, Yale University, New Haven, CT 06510, USA.; ^7^ National Center for PTSD, West Haven, CT 06477, USA.; ^8^ Wu Tsai Institute, Yale University, New Haven, CT 06511, USA.

## Abstract

Cortisol is known to promote memory for emotionally arousing experiences, yet the neural networks involved in enhancing these memories are unknown. Here, we combine pharmacological fMRI with an analysis approach to determine the dynamic brain network processes by which cortisol enhances the formation of emotional memories. We introduce dynamic connectome-based predictive modeling (dCPM) to identify high temporal resolution, whole-brain functional connectivity networks, and show that distinct networks can successfully predict trial-level arousal (involving salience network) and subsequent memory (involving visual, frontoparietal, and default mode networks). We find that, under cortisol, brain networks associated with both processing arousal and forming memories have more structural and functional overlap. Neural networks that predict arousal are more consistent and strongly engaged under cortisol, whereas networks that predict memory are more specialized to emotional memoranda. Together, these results reveal a dynamic process by which cortisol shifts whole-brain mechanisms to amplify emotional memories: Cortisol amplifies arousal-predictive networks while tuning memory-predictive networks to prioritize emotionally salient information.

## Teaser

The stress-related hormone cortisol alters dynamic brain networks predicting memory and arousal to promote emotional memories.

## INTRODUCTION

Stress powerfully influences what we learn and remember, prioritizing encoding of emotionally arousing and stress-relevant memoranda at the expense of neutral content (*1*–*10*). These benefits are related to increased levels of stress-related hormones such as glucocorticoids, as shown in both rodents [corticosterone (*11*–*13*)] and humans [cortisol (*14*–*19*)]. But how does the brain build emotionally salient memories under stress, and what role does cortisol play? Here, we combine pharmacological functional magnetic resonance imaging (fMRI) with advances in dynamic, whole-brain predictive modeling to define the neural mechanisms by which cortisol enhances the retention of individual emotional experiences.

When forming emotionally arousing memories, two key processes are engaged: detecting that an event is emotionally arousing and encoding that event for long-term storage. In principle, brain networks associated with both processes may be modulated by cortisol. With regard to arousal, the brain reallocates resources toward salience processing during stress, leading to network-wide changes in activity (*20*, *21*). Cortisol alone has been shown to accentuate effects of arousal-related neurotransmitters such as catecholamines throughout the brain (*22*). Behaviorally, there is evidence that cortisol can potentiate subjective arousal (*23*, *24*) and craving (*25*) [although these effects can be variable (*26*)]. Stress and cortisol can also promote learning of salient associations [both aversive (*27*) and appetitive (*28*)]. Together, these data suggest that cortisol may amplify arousal-related brain networks. Yet, the effects of cortisol on memory are not simply a matter of cortisol amplifying arousal: High arousal alone can impair memory (*29*), yet stress together with high arousal can enhance memory (*2*). Another possibility is that cortisol acts on networks associated with memory formation. Brain networks supporting emotional memory differ from those supporting memory for neutral events (*30*–*32*), and evidence from rodents shows that distinct brain regions promote emotional memories with cortisol (*12*). Thus, memory-predictive brain networks in humans may shift under cortisol, enabling preferential encoding of emotional (but not neutral) memoranda (*33*). Last, cortisol may act by shifting interactions between brain networks associated with memory and arousal (*34*, *35*). This is consistent with the finding that stress time-dependently acts on salience or executive control networks (*36*), dynamically altering intra- and internetwork functional connectivity (*34*, *35*, *37*). In the current study, we take a whole-brain perspective and develop analytic tools to investigate these dynamic processes.

Stress and cortisol act on a broad scale to influence processes throughout the brain. fMRI studies show that stress increases connectivity with the salience network (*38*) and frontal cortical areas (*39*), and recent rodent work demonstrates that stress alters cellular activity throughout the brain (*40*). These large-scale changes may be integral to glucocorticoid effects on the formation of emotional memories (*41*). Examining large-scale networks is also critical to disentangling cortisol effects on arousal processing from effects on memory encoding, as single regions can be involved in both processes. For instance, a large body of work has shown profound effects of cortisol on the amygdala [rodent studies (*12*, *19*, *42*) and human studies (*39*, *40*)] and interactions between amygdala and targeted regions such as the hippocampus [rodent (*43*–*45*) and human (*46*, *47*)] and medial prefrontal cortex [rodent (*48*) and human (*15*)]. As the amygdala is involved in both salience detection and memory formation (*32*), it is difficult to separate how cortisol affects neural mechanisms underlying these two processes. Thus, there is a need to separately define whole-brain networks associated with arousal and memory encoding and then quantify how connectivity within each network is altered when forming emotional memories under cortisol.

In addition to acting throughout the brain, the effects of stress and cortisol on the brain are known to change over time (*35*, *49*, *50*). Emotional arousal is also highly dynamic, as is the likelihood of remembering a given experience (*51*). Thus, the neural mechanisms by which cortisol influences emotional memory will also likely fluctuate. Ideally, this could be captured by quantifying connectivity on the scale of a single event, using this pattern to predict arousal and memory for that event, and then assessing how resultant networks differ with cortisol. Yet, commonly used techniques for computing connectivity typically do so over a period of minutes to predict an aggregate measure of behavior (*52*, *53*). Although we can estimate trial-level responses using univariate blood-oxygen level dependent (BOLD) analyses, and these estimates have been shown to predict later memory for a single event (*54*–*58*), this approach does not reveal how different regions across the whole brain work together to support memory. Critically, recent analytic advances have enabled dynamic measures of whole-brain connectivity to predict temporally fluctuating states (*59*, *60*). For example, dynamic brain connectivity can successfully predict moment-to-moment arousal (*61*) and engagement, which is in turn related to subsequent memory (*62*). By combining these advances with predictive modeling of arousal and memory, we can uncover the mechanisms by which cortisol dynamically potentiates the formation of emotional memories.

**Fig. 1.**
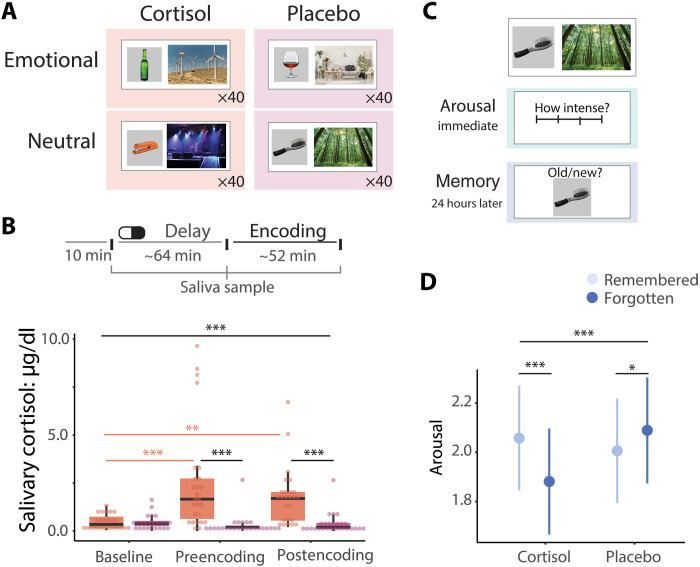
Experiment design and behavioral results. (**A**) Experiment design. Each participant encodes 40 trials of item-scene pair events under four different conditions. (**B**) Administration of hydrocortisone led to significant elevations in salivary cortisol throughout the encoding session (see also table S1). (**C**) Trial-level measurement of arousal and memory. Arousal is rated on a scale of 1 to 4 after viewing each event. Memory of the emotional and neutral objects is assessed 24 hours later with a recognition test (correctly recalled versus forgotten). (**D**) Under cortisol, more arousing objects were more likely to be remembered. ***P < 0.001, **P < 0.01, and *P < 0.05. Error bars = 95% confidence interval (CI).

**Fig. 2.**
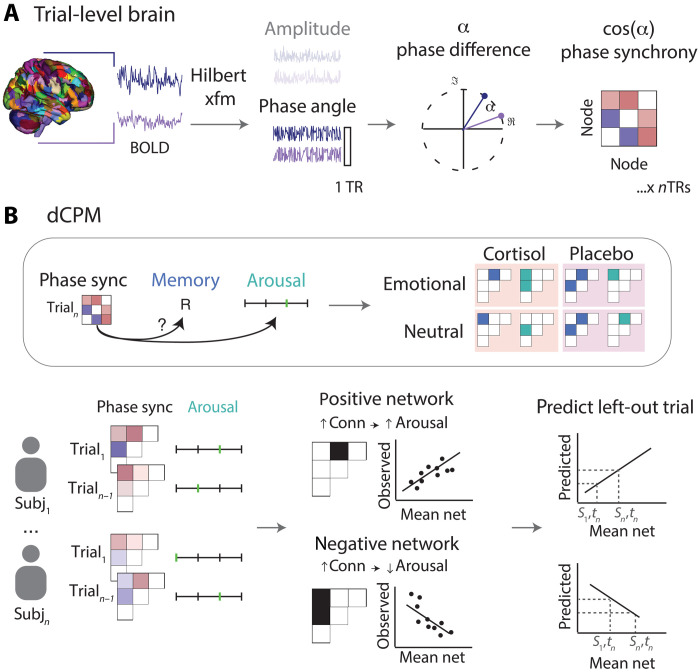
Analysis design. (**A**) Schematic of the trial-level phase synchrony extraction approach. (**B**) Schematic of dCPM. Edges that are significantly correlated with memory/arousal are selected. A linear model is then trained to predict memory/arousal on the left-out trial. This model is separately applied to all four study conditions (pill × emotionality) to yield four predictive networks. R, remembered.

In this study, we show that cortisol influences both memory and subjective arousal-predictive networks on a whole-brain basis with fine-grained temporal resolution. We used an experimental design in which each participant encoded unique events while undergoing fMRI under different pharmacological conditions (20 mg of oral hydrocortisone versus placebo) (Fig. 1, A and B) (*63*, *64*). During each scan session, these events were separated into two runs, one designed to evoke stronger emotional responses (alcohol/scene pairs) and one designed to evoke neutral responses (household object/scene pairs). With this design, we could derive trial-level measures of arousal (using participants’ subjective ratings during encoding) and memory (using item recognition performance on the following day; Fig. 1, C and D). We then leveraged dynamic connectivity techniques to quantify trial-level “snapshots” of whole-brain connectivity, measuring how synchronized the timing of BOLD activity is between pairs of brain regions (see Materials and Methods for details; Fig. 2A). With these trial-level brain and behavioral measures, we successfully introduced a machine learning approach, dynamic connectome-based predictive modeling (dCPM) (*59*), to define predictive networks as regions for which synchronized BOLD responses track trial-level memory and arousal. By defining these predictive networks under our different experimental conditions (Fig. 1A), we could determine the influence of cortisol on dynamic neural mechanisms underlying arousal and memory formation separately.

## RESULTS

### Effects of cortisol on emotional memory

We confirmed that administration of hydrocortisone led to increased salivary cortisol (pill: *F*_1,116_ = 28.76, *P* < 0.0001, pill × time point: *F*_2,114_ = 8.40, *P* = 0.0004 Fig. 1B).

As anticipated, we found that cortisol promoted memory for emotionally arousing information (Fig. 1D). We first examined whether remembered items differed in subjective arousal and how this was influenced by cortisol. This revealed a significant interaction (linear mixed effects model predicting arousal; pill × memory [remembered/forgotten]: *F*_1,4010_ = 28.76, *P* < 0.0001). Events that were remembered under cortisol were rated as subjectively more arousing (mean = 2.08, SD = 0.91) than events that were forgotten under cortisol (mean = 1.83, SD = 0.82; *t*_4010_ = 5.01, *P* < 0.001). Under placebo, we found the opposite pattern, with remembered items rated as subjectively less arousing (mean = 2.03, SD = 0.92) than those that were forgotten (mean = 2.05, SD = 0.97; *t*_4010_ = −2.38, *P* = 0.02). This interaction was particularly evident during emotional runs (run × pill × memory: *F*_1,4007_ = 7.09, *P* = 0.008). Thus, this dataset is well-suited to investigating the neural mechanisms by which cortisol prioritizes emotional encoding.

### Predicting trial-level arousal and memory with dynamic connectivity

**Fig. 3.**
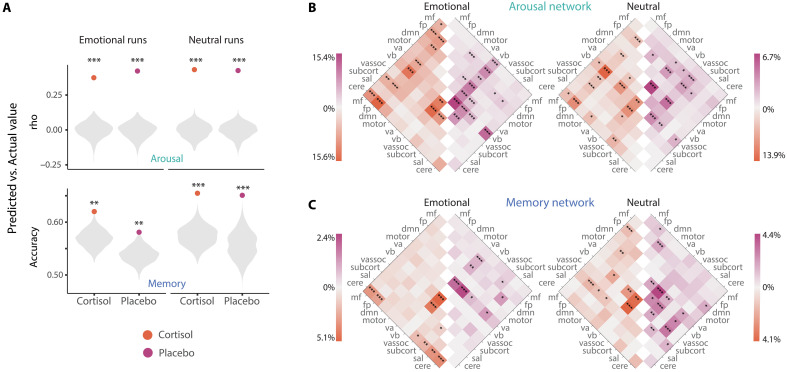
Successful prediction of arousal and subsequent memory using dynamic connectivity. (**A**) Model success (dots) compared to permutation testing (gray density plots). (**B**) Distribution of edges in arousal-predictive networks (positive subnetwork shown). The color of each cell represents the percentage of edges between pairs of predefined functional networks that are also in the predictive networks. Pairs for which more edges were included in the predictive network than expected by chance (determined by hypergeometric CDF; see details under the “Analysis” section) are marked with asterisks. (**C**) Distribution of edges in memory-predictive networks (positive subnetwork shown; for negative subnetworks, see fig. S1). *P < 0.05, **P < 0.01, and ***P < 0.001.

To understand the mechanisms by which cortisol potentiates emotional memory, we needed to identify brain circuits associated with arousal and memory formation, both of which are processes that fluctuate dynamically. To do so, we used dCPM to incorporate dynamic measures of connectivity and trial-level behavior. In this analysis, we divided the brain into nodes using an established atlas (*65*, *66*) and calculated phase synchrony to assess activity coherence between pairs of nodes (or “edges”) at each time point (Fig. 2A). We then assessed whether trial-level phase synchrony, or coherence, could predict trial-level arousal (thus identifying an arousal-predictive network) and subsequent memory (memory-predictive network), separately for each of the four runs (Fig. 2B). We used leave-one-trial-out cross-validation to determine the predictive power of each network and identified predictive networks as the edges that were selected on every leave-one-trial-out fold. Using this approach, we successfully predicted trial-level memory (accuracy ranging from 0.58 to 0.65) and arousal (*r* values ranging from 0.36 to 0.50) from dynamic phase coherence for each run and pill condition (Fig. 3A; all *P* < 0.005 via permutation testing, see table S2). This approach resulted in two subnetworks per model: One for which greater coherence was associated with higher performance (hereafter the positive subnetwork), and one for which greater coherence was associated with worse performance (negative subnetwork). Anatomical features of positive predictive subnetworks are visualized in Fig. 3 (B and C) (for negative subnetworks, see fig. S1).

### Cortisol changes which edges are involved in predictive networks

We next sought to quantify how cortisol altered the architecture (that is, which edges were included) of arousal-predictive and memory-predictive networks. First, we compared edges from predictive networks between cortisol and placebo conditions (i.e., the left and right halves of the heatmaps shown in Fig. 3 (B and C). The edges that comprised arousal-predictive networks were consistent under cortisol and placebo [edge overlap between pills: all *P* < 0.001, determined by hypergeometric cumulative distribution function (HCDF)]. However, memory-predictive networks varied, with no significant overlap in the edges that predicted emotional memory under cortisol compared to placebo (table S3). This analysis suggests that, although cortisol did not significantly change which edges comprised brain networks predicting arousal (regardless of emotionality), they did influence which brain networks predicted memory when encoding emotional stimuli.

**Fig. 4.**
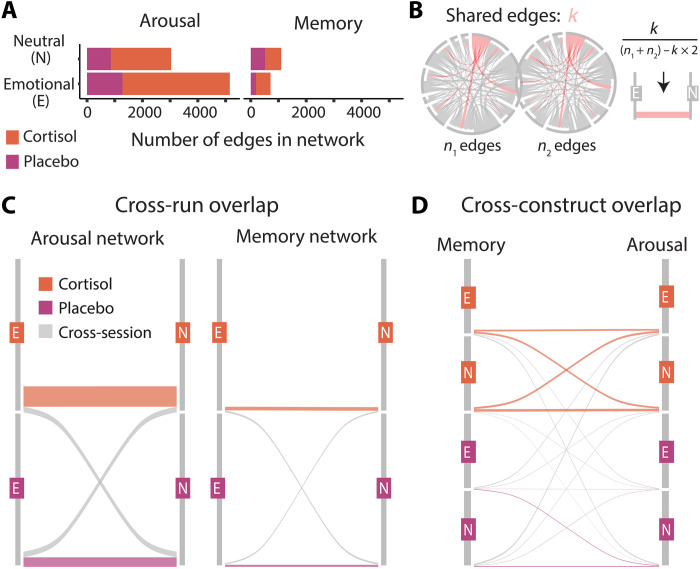
Overlapping edges between positive predictive networks under different pharmacological and affective conditions. (**A**) Size (number of edges) of positive predictive networks. (**B**) Schematic of data visualization showing overlap percentage between networks. (**C**) Overlap percentage between networks. In each plot, cortisol runs are shown at the top, and placebo runs are shown below. Orange lines indicate the percentage of overlap between cortisol runs, and pink lines indicate the percentage of overlap between placebo runs. Thicker lines correspond to a higher percentage of overlaps. Left: Common edges predicting arousal. Right: Common edges predicting memory. (**D**) Common edges predicting both arousal and memory. Note that for negative predictive networks, see fig. S2.

Given that we acquired two runs under each pill condition and thus built multiple predictive networks for each pill (Fig. 3A), we reasoned that cortisol could alter the extent to which those networks overlapped (indicative of more integration) or were distinct (indicative of more differentiation or specialization of the network to what was occurring in that particular run). To do so, we quantified the number of overlapping edges between the two runs acquired in each pill condition (controlling for the different sizes of these networks; Fig. 4A). Notably, we found that edges identified as part of predictive networks were generally consistent between runs obtained under the same pill, leading to a significant number of shared edges (Fig. 4, B and C, and table S4). This finding supports the reproducibility and robustness of these identified predictive networks.

Notably, the degree of consistency differed between arousal and memory-predictive networks. We found that arousal-predictive networks were more similar under cortisol than under placebo conditions [positive: *X*^2^(1,8186) = 58.53, *P* < 0.001; negative: *X*^2^(1,6586) = 61.08, *P* < 0.001; see fig. S3 for consistent edges under cortisol]. We did not find this pattern for memory-predictive networks. There was either no significant overlap between memory-predictive networks under cortisol (positive networks), or memory-predictive networks were more similar under placebo than cortisol [negative networks; *X*^2^(1,3122) = 8.4, *P* = 0.004]. As in our earlier analysis, these findings suggest that cortisol altered which brain networks predict memory. In contrast, cortisol increased the consistency or stability of the brain networks predicting arousal.

Last, we investigated whether there were common features between networks that predicted memory and arousal. That is, did cortisol lead memory and arousal-predictive brain networks to be more anatomically integrated (or overlapping)? This analysis revealed significantly more overlap between arousal-predictive and memory-predictive networks under cortisol (Fig. 4D). That is, subnetworks predicting memory and arousal were more similar to each other under cortisol compared to placebo (table S5). This pattern was particularly evident for positive subnetworks, with all pairs having greater overlap under cortisol than placebo (all *P* < 0.001) but was more variable for negative subnetworks.

Together, these anatomical comparisons suggest mechanisms by which cortisol dynamically enhances emotional memory. Under cortisol, arousal-predictive networks were more stable, and networks associated with memory and arousal were more integrated.

### Cortisol modulates predictive network engagement over time

**Fig. 5.**
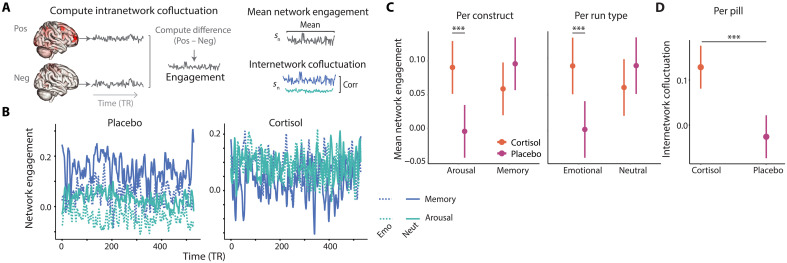
Predictive network engagement differs with cortisol and emotional content. (**A**) Schematic showing quantification of predictive network engagement. Warmer colors in brain images represent nodes with more connections in the predictive network. Right: Examples of how mean network engagement and internetwork cofluctuation are derived per subject. (**B**) Trace plots showing average network engagement across subjects for each run. (**C**) Differences in mean engagement (across run) based on predicted construct and run type. Error bars = 95% CI. (**D**) Differences in internetwork cofluctuation by pill. Error bars = 95% CI. ***P < .001.

In addition to influencing the edges that make up each network, cortisol could alter the extent to which arousal-predictive and memory-predictive networks were engaged during encoding. To test this, we first quantified the average cofluctuation within each of our identified positive and negative subnetworks. Then, for each construct (i.e., memory and arousal), we computed the difference between positive and negative intranetwork cofluctuation (Fig. 5A). This yielded a sense of how much, within each run, networks were engaged that predicted better memory and higher arousal (Fig. 5B). To separate the potential influence of anatomical overlap, all overlapping edges between memory and arousal networks in the same run were removed.

To determine how cortisol influenced engagement, we modeled network engagement as a function of construct (memory-predictive vs arousal-predictive), pill (cortisol vs placebo), and run (emotional vs neutral; Fig. 5C). We found a significant interaction between pill and construct (*F*_1,178_ = 20.56, *P* < 0.001), with greater engagement of arousal-predictive networks under cortisol [cortisol arousal versus placebo arousal: *t*(178) = 4.61, *P* < 0.001], whereas the engagement of memory-predictive networks did not differ between pills [cortisol memory versus placebo memory: *t*(178) = −1.81, *P* = 0.27]. We also found differences in network engagement based on pill and run type (*F*_1,178_ = 16.03, *P* < 0.001), with greater network engagement during emotional runs under cortisol compared to placebo [cortisol emotional versus placebo emotional: *t*(178) = 4.21, *P* < 0.001]. There were no significant three-way interactions. In a separate model without interaction terms, the only significant main effect was construct, such that memory networks, on average, were more engaged than arousal networks (*F*_1,179_ = 4.98, *P* = 0.027).

We next probed whether cortisol altered the temporal dynamics of how memory and arousal networks engaged with each other. That is, are these networks more synchronized under cortisol? We quantified moment-to-moment internetwork cofluctuation between predictive networks throughout the run (average engagement after removing shared edges shown in Fig. 5B). We then compared the degree of internetwork cofluctuation based on pill and run, finding that cofluctuation between memory-predictive and arousal-predictive networks differed between runs (neutral > emotional; *F*_1,76_ = 162.09, *P* < 0.001) and critically was higher under cortisol than placebo (*F*_1,76_ = 28.6, *P* < 0.001, two-way interactions not significant; Fig. 5D). That is, under cortisol, brain networks that predict arousal were more engaged, and brain networks predicting arousal and memory were more synchronized over time. As with the anatomical comparisons above, these results demonstrate that cortisol was associated with functional reorganization of brain networks underlying memory and arousal.

### Cortisol alters generalizability of predictive networks

The analyses above suggest an integration of memory and arousal predictive networks with cortisol. These networks share more features and (even after removing shared features) are more coordinated over time when individuals receive oral hydrocortisone compared to placebo. However, the directionality and functional impact of this reconfiguration remain unclear. That is, did cortisol change arousal networks to prioritize emotional memory formation? Or did cortisol shift memory networks to preferentially encode emotional content? The previous edge overlap analysis revealed that arousal-predictive networks were overall consistent, while memory predictive networks did not have significant overlap when encoding emotional stimuli between cortisol and placebo conditions. This suggests that, anatomically, it is more likely that cortisol altered memory-predictive networks to amplify the formation of memories for arousing experiences. However, it remains unclear whether the function of memory-predictive networks was likewise shifted.

**Fig. 6.**
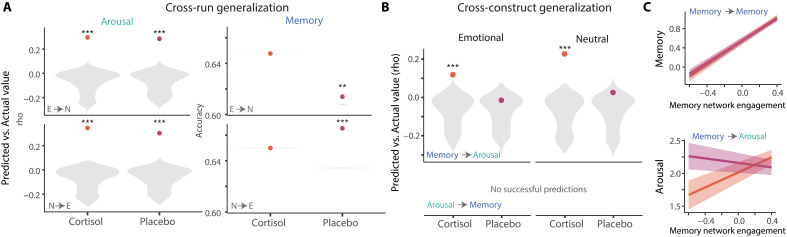
Generalization of predictions across runs and constructs. (**A**) Model trained on trial-level behavior from one run type (e.g., emotional) used to predict trial-level behavior in another run type (e.g., neutral). (**B**) Model trained on one type of trial-level behavior (e.g., memory) used to predict another trial-level behavior (e.g., arousal). Model success (dots) compared to permutation testing (density plots). (**C**) Illustration of generalization from memory to arousal. Trial-level engagement of the memory-predictive network is strongly related to memory performance (top) but only tracks arousal levels under cortisol (bottom). Shaded areas = 95% CI. **P < 0.01 and ***P < 0.001.

To address this question, we took a generalization approach to assess whether cortisol primarily repurposed memory-predictive or arousal-predictive networks. We first used dCPM to ask, within each pill condition, whether edges that were predictive on one run (e.g., emotional) would generalize to predict the same construct on the other run [e.g., neutral; Fig. 6A and table S6). Successful generalization would reveal that the same brain network supported this construct, regardless of whether emotional or neutral content was being encoded. We found successful generalization of arousal-predictive networks, but memory-predictive networks only generalized under placebo. These results suggest that cortisol recruited distinct memory-predictive brain networks when encoding emotional information.

We next conducted a more stringent test of generalization to examine this shift in memory-predictive networks under cortisol, and whether they indeed promoted encoding of emotionally salient content. Specifically, we asked whether edges associated with one construct (e.g., memory) could successfully generalize to predict the other construct (arousal; Fig. 6B and table S7). Successful generalization would show that a given brain network could predict both arousal and memory. This analysis was not successful under placebo conditions. We were also unable to use features associated with arousal to predict reliably the memory under both conditions.

Critically, we were able to generalize from memory to arousal under cortisol, with edges associated with memory yielding successful prediction of arousal (emotional run: rho = 0.08, *P* < 0.001; neutral run: rho = 0.23, *P* < 0.001). In other words, under cortisol, memory-predictive networks were sensitive to arousal, becoming more strongly engaged on more arousing trials (Fig. 6C). We note that this pattern was evident both in the emotional run (during which memory and arousal were correlated behaviorally) and the neutral run (during which these constructs were not correlated behaviorally). These results provide further evidence that cortisol was associated with integration of brain circuits supporting arousal and memory, indicating that memory-predictive networks may have been repurposed to prioritize emotionally salient information.

## DISCUSSION

Acute stress and the stress-related hormone cortisol alter memory for emotionally arousing events across species. Yet, our understanding of the mechanisms driving these benefits for human memory are limited. To understand these processes at a whole-brain and temporally dynamic level, we collected fMRI data while participants encoded emotionally salient and affectively neutral stimuli following hydrocortisone and placebo administration. Using a dCPM approach, we were able to explain how cortisol influenced emotionally salient encoding by identifying distinct brain networks predicting trial-level arousal and trial-level subsequent memory. The examination of the anatomical and functional properties of these dynamic predictive networks revealed key differences in how memory formation and arousal processing are instantiated in the brain and modulated by cortisol.

We found that cortisol boosted memory for items that participants perceived as emotionally salient. This is consistent with past findings that cortisol and stress around the time of encoding privilege memory for emotionally salient information (*1*–*10*, *16*, *17*) with neutral information not affected (*67*) or impaired (*68*). However, effects of stress and cortisol before encoding are complex (*7*, *69*), with some studies finding better memory for both neutral and emotional memoranda (*14*, *70*) or improvements in memory for neutral (*71*) [and not emotional (*72*)] items. Note that, in the current study, we did not find that cortisol was associated with category-level benefits for emotional memoranda. Instead, we showed that cortisol amplified memory for trials that the specific participant rated as subjectively arousing. This is consistent with evidence that memory benefits are associated with subjective changes in affect (*2*, *15*). As past studies have not consistently collected trial-level ratings, it is unclear whether variance in these subjective appraisals might contribute to mixed effects of cortisol on encoding. These results emphasize the need for participant-specific ratings of memoranda salience.

Beyond showing that brain responses are different when encoding emotional stimuli under cortisol or stress (*38*, *46*), our neuroimaging analysis approach enabled us to quantify whether cortisol-induced changes in emotional memory were driven by changes in brain networks related to memory, arousal, or both. Across analyses of anatomical overlap, network engagement, and generalization, our results revealed a consistent pattern. Cortisol influenced both arousal-predictive and memory-predictive brain networks when prioritizing emotional memory encoding but did so by different means. For arousal, cortisol led to more stable edges and stronger engagement of arousal-predictive networks throughout encoding. All arousal-predictive networks contained key nodes of the previously identified salience network, including anterior insula, midbrain, amygdala, inferior temporal cortex, and dorsal anterior cingulate cortex (*36*, *73*, *74*). These are also regions that have previously been implicated in attending to emotional stimuli under cortisol (*75*). Under cortisol, stable edges were largely in frontoparietal and visual networks. In addition, despite being statistically similar to the arousal-predictive networks defined under placebo, there were also macroscale differences in these networks under cortisol. For example, under cortisol, edges in arousal-predictive networks were mostly in frontoparietal and medial frontal networks, whereas under placebo, arousal edges were centered in visual network regions. These findings under cortisol are consistent with recent evidence that individuals in high arousal states also had greater medial frontal and frontoparietal connectivity (*76*). In addition, the arousal-predictive networks were substantially larger (containing more edges) under cortisol. Although we did not find that cortisol enhanced the subjective appraisal of arousal when viewing pictures (*23*, *24*), these findings accord with the idea that cortisol amplifies processing of salient information (*20*). Our observation of greater engagement of the arousal network under cortisol may also reflect increased brain function allocated to processing arousal (*77*, *78*). Together, these results suggest that cortisol may prioritize salient information, at least in part, through allocating more neural resources to arousal processing. Our findings are consistent with the idea that cortisol acts on the brain’s “salience” network and further suggest that salience potentiation may occur at an extended timescale, with continual engagement of arousal-predictive networks 1 hour post-oral hydrocortisone (*21*, *36*). They may also help explain why blocking cortisol at encoding can attenuate the effects of emotionality on memory (*79*). However, the role of cortisol specifically in stress-induced engagement of the salience network is unclear, with past reports attributing these effects to the adrenergic component of the stress response (*80*). Although we pharmacologically focused on cortisol in the current study, we did not specifically block the adrenergic system; thus, interactive effects of cortisol and adrenergic responses may have contributed to the engagement of the salience network. Further work is needed to test whether cortisol and adrenergic responses in isolation would similarly operate on arousal-predictive networks to potentiate salient encoding.

We also showed that cortisol was associated with changes in the neural mechanisms supporting memory. Memory-predictive networks became more specialized for emotionally salient content under cortisol. We found evidence for this interpretation anatomically by comparing the edges from our memory-predictive networks trained on neutral and emotional runs. Consistent with previous findings (*81*), edges in memory-predictive networks under placebo were mostly in salience, subcortical, default mode and visual networks. Under cortisol, these networks were distinct (no significant overlap between emotional and neutral runs); furthermore, networks trained on emotional runs were distinct between cortisol and placebo. Broadly speaking, these networks contained nodes known to be involved in the formation of memory for events, such as hippocampus and parahippocampal gyrus. Our evidence that these networks can be reorganized fundamentally when encoding under cortisol is consistent with recent time-varying connectivity studies showing that a successful encoding state was associated with systematic reconfiguration of a whole-brain network (*63*) and earlier work showing that distinct neural mechanisms support successful encoding under stress (*82*). One notable difference in the brain networks involved in emotional memory formation under cortisol concerns the involvement of the cerebellum, adding to burgeoning evidence that the cerebellum is involved in emotional processing and stress (*83*–*85*) and recent findings that hydrocortisone, together with an adrenergic agent, increases cerebellar connectivity (*86*). Together, these findings are consistent with the proposal that stress promotes a “memory formation mode”(*87*) and further suggest selective tuning under cortisol to prioritize emotionally salient memoranda.

In addition to separately modulating neural networks associated with arousal and memory, elevated cortisol at encoding also led to greater integration between these circuits. Anatomically, we found more overlapping edges between memory-predictive and arousal-predictive networks under cortisol. Even after removing these overlapping edges, we found that cortisol led to more synchronized temporal dynamics between these networks. These internetwork cofluctuations are increasingly recognized as providing important insight into brain organization and dynamics associated with stress and emotional memory (*88*, *89*).

Beyond these spatial and temporal alterations, we found that cortisol promoted greater functional integration between these constructs. This generalization analysis also revealed important insights into directionality: that is, were memory-predictive networks tuned to carry information about arousal, or were arousal-predictive networks modulated to subserve successful encoding? We found evidence for the former, as memory-predictive networks generalized to predict arousal under cortisol, but no evidence for the latter. Considering these findings in tandem with effects on each predictive network separately, our data indicate that cortisol alters memory-predictive circuits, tuning them to prioritize encoding of emotionally arousing stimuli and bringing them closer to arousal-predictive circuits. Although further studies are needed to test this framework, these results provide key insights into the internetwork dynamics by which cortisol tunes successful emotional memory formation.

We also note limitations to the current design. First, although targeting cortisol provides specific mechanistic insights, there is a need to investigate other components of the acute stress response (*90*). While acute stress has also been shown to enhance emotional memory (*1*, *2*, *5*–*7*), it is possible that other components of the physiological cascade associated with the body’s stress response would lead to distinct neural mechanisms (*80*). Second, this study focused on appetitive arousing stimuli. We chose to use these images as they are salient, are behaviorally relevant (*63*, *91*), and have motivational value (see Materials and Methods). Although the use of these images provides an important extension of past acute stress studies showing similar effects on negative high-arousal stimuli (*2*, *6*), the computations underlying memory for positive events may differ from those for negative events (*92*, *93*). Thus, further work is needed to examine the ways in which cortisol modulates memory-predictive networks to promote negative memories and salient images from other categories. Third, the level of subjective arousal induced by these stimuli was moderate. This raises the possibility that brain networks supporting arousal and memory may be differentially modulated by cortisol when encoding highly arousing stimuli (*94*). Last, we did not assess physiological markers of stimulus-evoked arousal. Previous work has shown that effects of cortisol on memory for arousing content are tightly linked to adrenergic responses (*12*, *19*), and subjectively arousing stimuli have also been shown to evoke physiological correlates of adrenergic responses (*95*). Nevertheless, as it is possible that different brain networks would track physiological and subjective arousal, there is a need for future work to examine how interactions between physiological arousal networks and memory-predictive networks are modulated by cortisol.

The analysis innovations presented here may be applied broadly to address key questions in cognitive neuroscience. At a practical level, dCPM amplifies statistical power by predicting behavior per individual trial (leading to *n*_subjects_ × *n*_trials_ data points) rather than averaged behavior (*n*_subjects_ data points only). In addition to the statistical benefits, dCPM also provides a critical increase in temporal resolution. For example, in the memory domain, dCPM enables us to transition beyond average static connectivity/average memory performance throughout encoding (*96*). Furthermore, as with other whole-brain predictive modeling techniques, dCPM allows for an understanding of whole-brain processes beyond targeted regions of interest while maintaining this high temporal resolution. With stress, changes in larger-scale brain networks are likely necessary to understand alterations in processes such as executive control and attentional vigilance (*20*) and shifts in how memory is encoded (*36*). Critically, dCPM allows for the definition of whole-brain predictive networks within the context of an experiment (rather than using externally defined networks). This provides an opportunity to capture alterations in which parts of the brain are involved in each predictive network under different conditions or between different groups [see also (*65*)] and providing an opportunity to quantify the consistency and generalizability of predictive networks. Together, these innovations will provide key whole-brain and temporally precise insights into how the brain supports cognition and affect.

In summary, these findings provide a framework for understanding how cortisol amplifies emotional memory. We introduce tools to quantify whole-brain and temporally dynamic processes supporting emotional memory formation under cortisol, adding to emerging work leveraging dynamic connectivity to understand fluctuations in affective human behavior (*97*). Ultimately, our results take an important step toward uncovering the adaptive features of the acute stress response and the neural mechanisms by which cortisol enhances emotional memories.

## MATERIALS AND METHODS

### Participants

All participants provided written informed consent before participation, and all procedures were approved by the Yale Institutional Review Board (HIC 2000026404). A total of 27 participants completed two scans each, one with hydrocortisone and one without hydrocortisone. Each scan session was followed 24 hours later by a memory retrieval session. Because of a technical error, one participant did not have usable data from one retrieval session. Thus, the 26 participants who had full fMRI and retrieval data acquired were included in the present analysis (fig. S4; 52 scans, 4160 trials total; 42.3% female: mean age = 27.62 [SD = 5.56]).

### Pill administration and cortisol measurement

Hydrocortisone administration and randomization were described previously (*64*). Pills were administered approximately 1 hour before first encoding run (mean = 64.3 [SD = 9.2] minutes) to allow for pill metabolism and target significant elevations in central cortisol throughout encoding [consistent with (*16*, *72*]. Encoding lasted less than 1 hour (mean = 52.1 [SD = 8] minutes). The order in which participants received hydrocortisone or placebo was counterbalanced across participants.

To measure hydrocortisone efficacy, participants provided saliva samples throughout the encoding sessions (baseline, pre-encoding, and postencoding). Samples were collected using Starstedt Salivette Tubes and processed by the Yale Center for Clinical Investigation using radioimmunoassay. Participants were unaware of which pill they had received and had no significant changes in overall affective state [see (*64*) for further details].

### Encoding and retrieval tasks

During each scan, participants encoded trial-unique associations between photographs of objects and scenes. Scans were divided into two runs, one in which the objects were emotionally salient (alcoholic beverages, *N* = 40 per participant; see the “Stimuli” section for details) and one in which they were neutral household objects (*N* = 40 per participant). On every trial, participants viewed an object and scene pair (5 s) and rated how they felt when imagining the object interacting with the scene (question: “Choose if you felt happy, neutral, or unhappy”; 2 s per rating), including how intense their feelings were [subjective arousal; question: “Rate how intensely you felt that way” with options from 1 to 4, end points labeled “Not at all intense” (1) and “Extremely intense” (4)].

Participants completed this process twice, once receiving 20 mg of hydrocortisone before encoding and once receiving a visually identical placebo tablet. This resulted in a 2 × 2 design combining emotionality (neutral versus emotional runs) and pharmacology (cortisol versus placebo tablets). Run order was counterbalanced across participants, with participants completing the same run order (e.g., emotional and then neutral) under both placebo and cortisol conditions, thus avoiding potential interactive effects of stimulus order (*98*) and pill condition.

The day after encoding, participants returned to complete an item recognition task (outside the scanner) in which they viewed 80 images of objects viewed during encoding and 80 category-matched novel objects (3 s each). On each trial, participants indicated whether they viewed the item during encoding (old) or not (new; 2-s response window), leading to trial-level memory scores of correct (1) or incorrect (0). Memory was tested in the same order as encoding (i.e., if emotional stimuli were encoded first, then they were also retrieved first).

Only trials with valid memory scores or arousal ratings are included in the corresponding analyses for building memory and arousal networks. For detailed exclusion criteria, please see the sections for each analysis.

### Stimuli

Object and alcohol images are described in greater detail in (*64*). We chose to use images of alcohol for our emotional stimuli as we have previously shown that images of alcohol are behaviorally relevant, have motivational value, and are rated as subjectively arousing (*99*). All participants in the study were over 21 years old and were social drinkers of alcohol [for more details about alcohol use, see (*63*)].

To validate that images from alcohol and household object categories would evoke different emotional responses, an online cohort (*N* = 165) rated a large corpus of *N* = 398 images on affective features including valence (Self-Assessment Manikin image scale; 1 = “unhappy” to 5 = “happy”) and arousal (1 = “calm” to 5 = “excited”) (*100*) and perceptual features such as familiarity and visual complexity. These ratings validated that alcoholic beverages elicited stronger emotional responses than neutral household objects, including higher levels of arousal [*t*(396) = 30.64, *P* < 0.001].

We used these ratings to pseudorandomly select a subset of *N* = 320 images per participant, such that (i) there were no significant differences in ratings between sessions (i.e., content encoded under cortisol versus placebo), (ii) there were no significant differences in ratings between images chosen to be encoded and those presented as lures during the recognition memory test, and (iii) that sets of alcohol and object images did not differ significantly in perceptual features.

### Scan parameters and preprocessing

Data were acquired across Siemens 3 T Prisma scanners using a 64-channel coil. Parameters were the same across all scanners. Every participant completed both MRI sessions in the same scanner. Functional images were collected using an echoplanar imaging (EPI) sequence [1-s TR, 30-ms TE, 75 axial slices, 2-mm^3^ voxels, 55° flip angle, multiband factor of 5, and a field of view (FOV) of 220 × 220]. High-resolution T1-weighted 3D MPRAGE was used for anatomical images (2400-ms TR, 1.22-ms TE, 208 sagittal slices, 1-mm^3^ voxels, 8° flip angle, and an FOV of 256 × 256).

Initial preprocessing steps followed those described in (*64*). In brief, after ensuring that motion was below our a priori threshold (<1.5-mm mean frame-to-frame displacement), functional data were skull-stripped [BET; (*101*)], prewhitenened, and high-pass filtered. Motion (6 degrees of freedom) and white matter time series were regressed out, along with their temporal derivatives and stick function regressors for nonlinear motion using FSL’s FEAT (*102*). No smoothing was applied.

Next, we took the residuals of this general linear model and band-pass–filtered them to 0.008 to 0.12 Hz in preparation for Hilbert transformation (*103*). We then aligned functional data to standard space by concatenating transformations from functional to structural (boundary-based registration) and from structural to MNI space. Last, we computed the mean time series across voxels from each of 368 nodes in the Shen-368 atlas (*104*) plus an additional nine nodes from regions associated with the stress response (*65*, *66*).

#### Analysis

Analyses were conducted in Python 3.12.4 and MATLAB 9.14.0.2337262 (R2023a). Linear mixed effects models (LMEs) were run in R 4.3.1 using the nlme package. For all LMEs, separate models were run with and without interaction terms to estimate main effects.

### Behavior analyses

To validate the effect of hydrocortisone pill on salivary cortisol, we fit an LME predicting salivary cortisol as a function of pill and time point with subject as a random effect, running separate models to identify main effects and interactions.

To assess the influence of pill and run type on memory and arousal, we built models separately predicting memory and arousal and testing for the relationship between these constructs. Trial number was always included as a predictor in the models to control for order effects with subject as a random effect.

For arousal, we first fit an LME on arousal with run (emotional versus neutral) and pill (cortisol versus placebo) as predictors to identify any main effects. We then fit a separate LME allowing interactions between run and pill. For memory (for which values could only be 1 = remembered or 0 = forgotten), we fit binomial-generalized LMEs predicting memory as a function of run and pill, running separate models with and without interactions.

To assess cortisol effects on memory and arousal, we fit an LME predicting arousal as a function of run, memory, and pill. One version included only a two-way interaction (run and memory), and one included three-way interactions.

### Calculating dynamic connectivity

To derive a dynamic measure of connectivity, we used phase synchrony to assess activity coherence between two nodes at each time point. Phase synchrony was used because it allows time resolution to be as precise as 1 TR (1 s in this design). If the time series from two nodes have high phase synchrony, then this indicates that their signal fluctuates similarly.

To calculate phase synchrony, we first used Hilbert transformation to compute time-series values to phase angle per TR and node. Then, between each pair of nodes, phase synchrony was calculated as the cosine of the difference between phases from each node. This analysis resulted in a 377 × 377 phase synchrony matrix per time point during encoding.

### Dynamic connectome-based predictive modeling

In our first analysis, we tested whether dynamic phase synchrony could predict trial-level item memory and arousal ratings with dCPM. Models were built separately for each fMRI run (40 trials each × 26 participants = 1040 trials per model) to predict trial-level arousal ratings and subsequent memory. In each run separately, trials with missing behavior values (memory score or arousal rating) were excluded from analysis.

As inputs to each model, we used trial-averaged phase synchrony between all pairwise combinations of 377 nodes. To account for the hemodynamic lag, we shifted the data 5-s forward and then calculated averaged phase synchrony across a 10-s window. On each iteration of the model, we left out one trial across all participants. We then selected features by correlating edges from the averaged phase synchrony matrices with behaviors from the remaining trials. Edges that correlated with behaviors at our threshold (*P* < 0.01) are selected and summed to build a logistic regression model predicting memory or a linear regression model predicting arousal. We then tested the model to predict behavior on the left-out trial. The performance was assessed using Pearson’s correlation (for arousal) or accuracy (for memory) between predicted and actual behavior, with permutation testing (1000× iterations of building the model with randomly shuffled data) to determine significance.

### Network anatomy comparisons

In addition to determining model success, we identified the edges that comprise predictive networks. We define these as the edges that are selected on every leave-one-trial-out fold (*65*). For each dCPM model, two predictive subnetworks were defined: one in which stronger phase synchrony corresponds to higher behavioral metrics (known as positive subnetworks) and one in which stronger phase synchrony corresponds to lower behavioral metrics (known as negative subnetworks).

To characterize the anatomy of memory- and arousal-predictive networks, we first grouped our 377 nodes into 10 predefined functional networks (*65*). Then, each edge that was identified as part of one of our predictive networks (i.e., an edge that was selected on every leave-one-trial-out fold) could be described as belonging to a particular functional network pair. We calculated the percent of edges within each functional network pair that were identified as edges in our positive and negative predictive subnetworks. We quantified whether this percent was statistically significant (that is, whether more from a given subnetwork pair belonged to our predictive subnetwork than would be expected by chance) using an HCDF (for details, see the “Anatomical overlap between networks” section).

### Anatomical overlap between networks

We computed the percentage of edges that overlapped between two predictive networks by dividing the number of overlapping edges by the number of edges in the smaller network. The statistical significance of this overlap was determined using an HCDF, which calculates the probability of obtaining *x* (number of overlapping edges) of *y* possible items (number of edges in one network) with *n* drawings (number of edges in another network) from an *M*-item population (total number of edges in matrix, 70,876). For example, to determine whether 25 overlapping edges between memory-predictive networks identified under cortisol was a statistically significant degree of overlap (neutral run: 582 total edges; emotional run, 521 edges), we calculated the probability of obtaining 25 of the 582 edges with 521 drawings from a population of 70,876 (all possible edges).

To compare the degree of overlap between two sets (e.g., overlap between networks defined under cortisol compared to overlap between networks defined under placebo), we used Chi-square tests. Contingency tables included the normalized overlap of one network pair (e.g., A and B) and the comparison network pair (C and D), where *k* reflects the number of overlapping edges and *n* is the total edges in the network (visualized in Fig. 4). Significant results indicate higher overlap between one pair of networks (e.g., A and B) than the other pair of networks (C and D).

### Network engagement and cofluctuation

After extracting phase synchrony per time point for all edges in each predictive network, we quantified two measures of dynamics throughout the scan. First, network engagement was defined as the difference between positive and negative subnetwork synchrony (*66*). Thus, positive values indicate that the positive subnetwork is more in sync than the negative subnetwork, indicating that engagement is biased toward boosting behavior. To assess whether network engagement differed by run and pill for each construct (memory and arousal), we computed the average network engagement per subject and run. We then fit an LME predicting engagement as a function of run (emotional versus neutral), pill (cortisol versus placebo), and construct (emotional versus neutral) with subject as a random effect. We separately fit other LMEs allowing for two-way interactions between the three predictors (run, pill, and construct) and an LME with a three-way interaction.

Second, we quantified internetwork cofluctuation as the correlation between engagement of two networks. To assess whether this coupling was influenced by emotionality and cortisol, we computed internetwork cofluctuation between memory and arousal-predictive networks per subject and run. Again, we fit an LME, here predicting internetwork cofluctuation as a function of run and pill with subject as a random effect. We also fit an LME allowing two-way interaction between run and pill. Note that overlapping edges between memory and arousal-predictive networks under each run were removed to avoid confounds for this analysis.

### Network generalization

We took a generalization approach to test whether there was functional overlap in the predictive power of these networks. We first asked whether edges that were predictive on one run (e.g., emotional) would generalize to predict the same construct on the other run from the same pill condition. To do this, we modified the CPM approach to identify edges based on one run but relate brain and behavior from the other run. For example, to test whether networks would generalize from neutral to emotional runs, we performed the following steps: (i) identify edges that significantly correlated with memory in the neutral run (*P* < 0.01) as in the original models above (i.e., leaving out a single neutral “test” trial and selecting edges that correlated with the “training” trials); (ii) compute responses during the emotional training trials from the neutral-selected edges (leaving out single emotional “test” trial); (iii) build a logistic regression model relating these emotional responses to memory; (iv) apply this model to the left-out emotional trial to predict memory; and (v) repeat process, leaving out each trial one at a time. Thus, this approach tested whether edges related to memory during a neutral run could successfully generalize to predict memory on an emotional run.

We then assessed functional overlap by assessing whether dynamic metrics of brain signal that could predict one construct (e.g., memory) could also predict another construct (e.g., arousal). We used the same approach as described above. For example, edges that were significantly associated with memory were selected to build a model and predict arousal on the left-out trial. Thus, edges selected on every fold were the same as those selected when building the “memory network” described earlier, but we could assess the utility of those same edges for predicting another construct. As above, model significance was confirmed via permutation testing.
