# Supplementary material for: Dynamic brain mechanisms supporting salient memories under cortisol
Source: Sci Adv. 2025 Dec 10;11(50):eadz4143. doi: 10.1126/sciadv.adz4143 (PMC12693973; doi:10.1126/sciadv.adz4143)
Supplement: Supplementary file 1 — Figs. S1 to S4 Tables S1 to S7 [file sciadv.adz4143_sm.pdf]

Science Advances
AAAS

Supplementary Materials for

Dynamic brain mechanisms supporting salient memories under cortisol

Yuye Huang *et al.*

Corresponding author: Elizabeth V. Goldfarb, elizabeth.goldfarb@yale.edu

Sci. Adv. **11**, eadz4143 (2025)
DOI: 10.1126/sciadv.adz4143

This PDF file includes:

Figs. S1 to S4
Tables S1 to S7

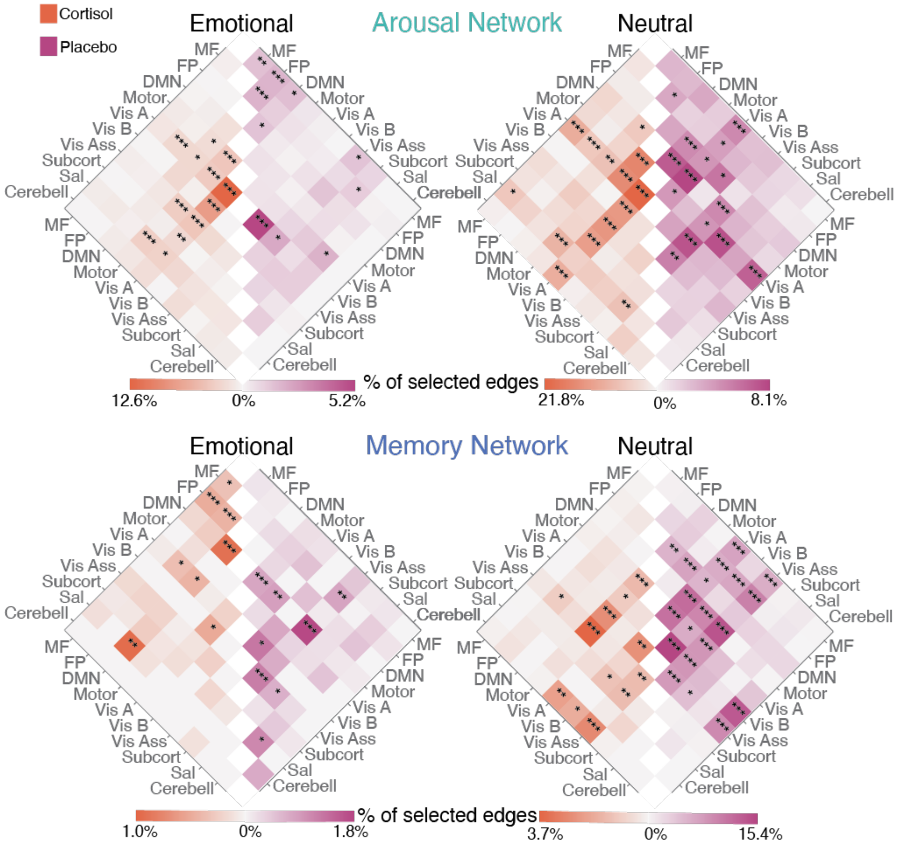

Fig. S1.

Distribution of edges in negative memory-predictive and arousal-predictive networks (negative subnetworks). The color of each cell represents the percentage of edges between pairs of predefined functional networks that are also in the predictive networks. Pairs for which more edges were included in the predictive network than expected by chance (determined by hypergeometric CDF) are marked with asterisks.

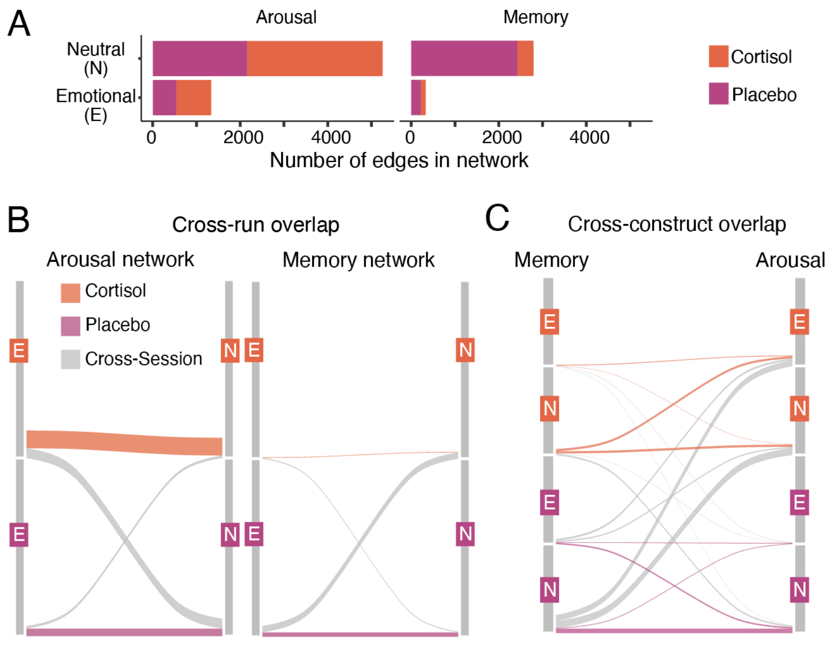

Fig. S2.

Overlapping edges between negative predictive networks under different pharmacological and affective conditions. (A) Size (number of edges) of negative predictive networks. (B) Overlap percentage between networks. In each plot, cortisol runs are shown at the top and placebo runs are shown below. Orange lines indicate the percentage of overlap between cortisol runs, pink lines indicate the percentage of overlap between placebo runs. Thicker lines correspond to higher percentage of overlap. Left, common edges predicting arousal; below, right edges predicting memory. (C) Common edges predicting both arousal and memory. Note: for positive predictive networks, see main text Figure 3.

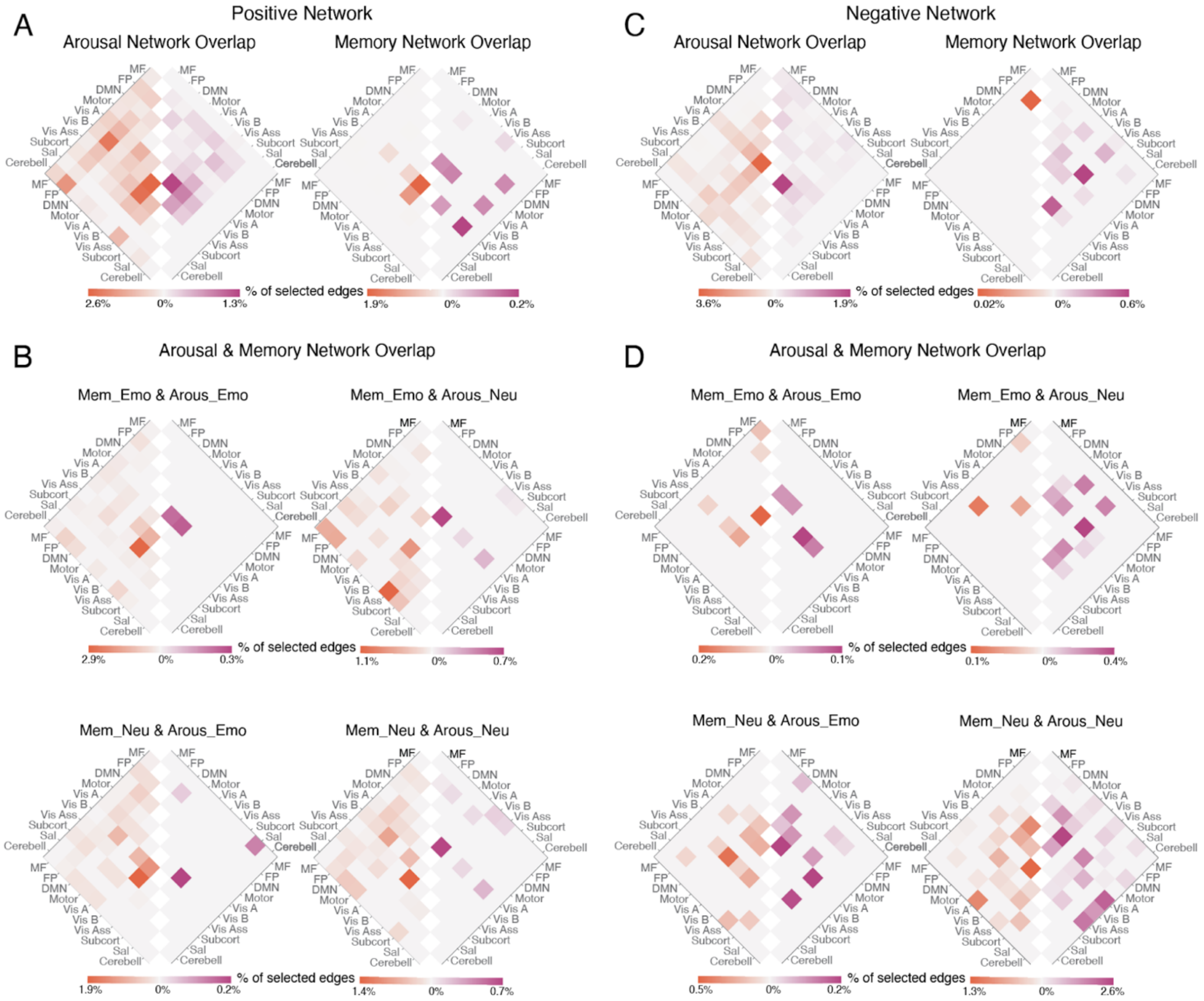

Fig. S3.

Distribution of edges in overlapping networks. Positive, left; negative, right. (A,C) Distribution of overlapping edges in arousal-predictive networks (overlap between arousal network in emotional run and neutral run) and overlapping edges in memory-predictive networks (overlap between memory network in emotional run and neutral run) . The color of each cell represents the percentage of edges between pairs of predefined functional networks that are also in the predictive networks. (B,D) Distribution of overlapping edges between memory and arousal predictive networks.

<insert page break here>

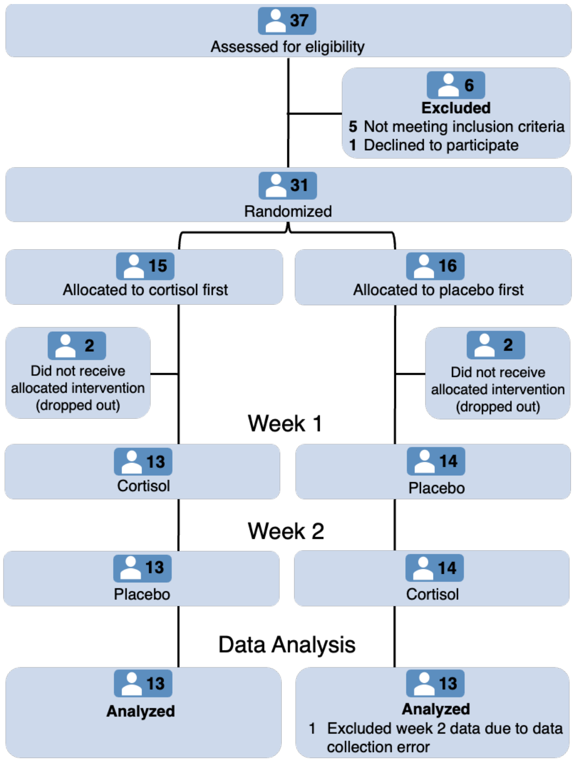

Fig. S4.
CONSORT diagram

Pill	Timepoint	Cortisol level (μg/dL)
Hydrocortisone	Baseline	M = .47 [SD = .38]
	Pre-Encoding	2.57 [2.82]
	Post-Encoding	1.86 [1.49]
Placebo	Baseline	.45 [.39]
	Pre-Encoding	.32 [.52]
	Post-Encoding	.37 [.53]

Table S1.
Cortisol levels per saliva sample

Behavior	Pill	Run type	Prediction success (overall)	Prediction success (pos network only)	Prediction success (neg network only)	Pos Edges (N)	Neg Edges (N)
Memory	Cortisol	Emotional	acc = .62, *p* = 0.001	acc = .66, *p* < 0.001	acc = .63, *p* = 0.001	521	111
		Neutral	acc = .65, *p* < 0.001	n.s.	acc = .63, *p* = 0.021	582	371
	Placebo	Emotional	acc = .58, *p* = 0.003	acc = .58, *p* = 0.027	n.s.	192	217
		Neutral	acc = .65, *p* < 0.001	acc = .63, *p* < 0.001	acc = .63, *p* < 0.001	515	2423
Arousal	Cortisol	Emotional	rho = .36, *p* < .001	rho = .21, *p* < .001	rho = .15, *p* = .002	3873	796
		Neutral	rho = .43, *p* < .001	rho = .20, *p* < .001	rho = .19, *p* < .001	2180	3099
	Placebo	Emotional	rho = .43, *p* < .001	rho = .17 *p* < .001	rho = .13, *p* < .001	1279	537
		Neutral	rho = .50, *p* < .001	rho = .18, *p* < .001	rho = .18, *p* < .001	854	2154

Table S2.

Results of dCPM predictions of trial-level memory and arousal
Prediction success represents network prediction performance, separated for overall (positive and negative), positive, and negative networks. Because trial-level memory performance is binary, memory models are evaluated by prediction accuracy (acc). Arousal models are evaluated by correlation between predicted and actual value (rho). p-values obtained from permutation testing.

		Emotional		Neutral	
**Network**	**Direction**	**# shared edges (Cort v Plac)**	**HCDF**	**# shared edges (Cort v Plac)**	**HCDF**
Memory	+	3	$p = .054$	20	$p < .001$
	-	1	$p = .046$	86	$p < .001$
Arousal	+	162	$p < .001$	116	$p < .001$
	-	19	$p < .001$	447	$p < .001$

Table S3.

Effects of run emotionality on overlapping edges. Comparisons between networks predicting the same construct. HCDF = hypergeometric cumulative distribution function.

Network	Direction	Cortisol: # shared edges (Emo v Neut)	Cortisol: HCDF	Placebo: # shared edges (Emo v Neut)	Placebo: HCDF		Cortisol vs Placebo? (Chi sq)
Memory	+	25	*p* < .001	9	*p* < .001		$X^2(1,1776) = 1.88$, *p* = .17
	-	1	*p* = .115	62	*p* < .001	^	$X^2(1,3059) = 8.64$, *p* = .003
Arousal	+	702	*p* < .001	123	*p* < .001	*	$X^2(1,7361) = 71.24$, *p* < .001
	-	350	*p* < .001	107	*p* < .001	*	$X^2(1,6129) = 70.34$, *p* < .001

Table S4.

Effects of cortisol on overlapping edges. Comparisons between networks predicting the same construct. HCDF = hypergeometric cumulative distribution function. ^placebo > cortisol; *cortisol > placebo

Networks	Direction	Cortisol		Placebo			Cortisol vs Placebo? (Chi sq)
		# shared edges	HCDF	# shared edges	HCDF		
Emotional: Memory vs. Arousal	+	97	*p* < .001	2	*p* = .675	*	$X^2(1,5766) = 27.95$, *p* < .001
	-	6	*p* < .001	4	*p* = .025		$X^2(1,1651) = 0.001$, *p* = .98
Neutral: Memory vs. Arousal	+	86	*p* < .001	13	*p* = .004	*	$X^2(1,4032) = 18.18$, *p* < .001
	-	80	*p* < .001	212	*p* < .001	^	$X^2(1,7755) = 32.15$, *p* < .001
Emotional: Memory vs. Neutral: Arousal	+	62	*p* < .001	7	*p* = .002	*	$X^2(1,3678) = 10.48$, *p* = .001
	-	4	*p* = .537	33	*p* < .001	^	$X^2(1,5544) = 31.85$, *p* < .001
Neutral: Memory vs. Emotional: Arousal	+	108	*p* < .001	3	*p* = .984	*	$X^2(1,6138) = 37.05$, *p* < .001
	-	26	*p* < .001	17	*p* = .567	*	$X^2(1,4084) = 21.23$, *p* < .001

Table S5.
Effects of cortisol on overlap between arousal- and memory-predictive networks. HCDF = hypergeometric cumulative distribution function. ^placebo > cortisol; *cortisol > placebo; gray = n.s.

Behavior	Pill	Trained on	Tested on	Prediction success (overall)	Prediction success (pos network only)	Prediction success (neg network only)
Memory	Cortisol	Neutral	Emotional	n.s.	n.s.	n.s.
		Emotional	Neutral	n.s.	n.s.	n.s.
	Placebo	Neutral	Emotional	acc = .67, $p < 0.001$	acc = .64, $p = 0.002$	n.s.
		Emotional	Neutral	acc = .61, $p = 0.002$	n.s.	n.s.
Arousal	Cortisol	Neutral	Emotional	rho = .29, $p < .001$	rho = .21, $p < .001$	rho = .08, $p < .001$
		Emotional	Neutral	rho = .35, $p < .001$	rho = .13, $p < .001$	rho = .18, $p < .001$
	Placebo	Neutral	Emotional	rho = .30, $p < .001$	rho = .13, $p < .001$	rho = .07, $p = .006$
		Emotional	Neutral	rho = .34, $p < .001$	rho = .08, $p < .001$	rho = .15, $p < .001$

Table S6.
Generalization of dCPM predictions between runs. As in Table S1, prediction success represents network prediction performance, separated for overall (positive and negative), positive, and negative networks. Because trial-level memory performance is binary, memory models are evaluated by prediction accuracy (acc). Arousal models are evaluated by correlation between predicted and actual value (rho). p-values obtained from permutation testing.

Trained on	Tested on	Pill	Run type	Prediction success (overall)	Prediction success (pos network only)	Prediction success (neg network only)
Arousal	Memory	Cortisol	Emotional	n.s.	n.s.	n.s.
			Neutral	n.s.	n.s.	n.s.
		Placebo	Emotional	n.s.	n.s.	n.s.
			Neutral	n.s.	n.s.	n.s.
Memory	Arousal	Cortisol	Emotional	rho = .13, $p < .001$	rho = .11, $p < .001$	n.s.
			Neutral	rho = .22, $p < .001$	rho = .08, $p < .001$	rho = .12, $p < .001$
		Placebo	Emotional	n.s.	n.s.	n.s.
			Neutral	n.s.	n.s.	n.s.

Table S7.
Generalization of dCPM predictions across constructs. As in Table S1, prediction success represents network prediction performance, separated for overall (positive and negative), positive, and negative networks. p-values obtained from permutation testing.
